# Notes and News

**Published:** 1896-07-25

**Authors:** 


### Notes and News.

(Collected and Communicated.)

The Order of St. Michael and St. George has been presented to Surgeon-Lieutenant-Colonel Blennerhassett, and that of the Indian Empire to Surgeon-Lieutenant-Colonel Haslett Browne.

At Kosheh the cholera has again made itself evident, and fatigue parties are engaged in constructing a new hospital in the desert, to which the patients will be removed, and the huts they occupy at present burnt.

Mr. Justice Vaughan Williams presided at the annual distribution of medals, prizes, and certificates, and the presentation of scholarships in connection with the Charing Cross Hospital Medical School.

Halifax is making great preparations for the visit of the Duke and Duchess of York to open the new infirmary on Saturday. The sum of £80,000 has been raised towards building the hospital, and £20,000 is still wanting to defray the cost.

The York wing at the Royal Sheffield Hospital is ow completed. It will be remembered that the foundation stone was laid by the Duke and Duchess of York in 1894. The cost of the additions to the hospital have cost nearly £50,000. The new out-patients' department has been provided, besides extra wards and offices. Electric lighting and numerous modern improvements have been introduced.

Sir William MacCormac has been elected President of the Royal College of Surgeons. He holds the post of consulting surgeon at St. Thomas's Hospital, where he is also Emeritus Lecturer in Clinical Surgery. He was elected examiner of the Royal College of Surgeons in 1887, and was twice vice-president. Sir William MacCormac is well known as an ambulance surgeon, and has had some interesting campaigning experiences. He has made some valuable contributions to medical literature and performed several remarkable and important surgical operations.

H.R.H. the Duchess of Connaught will lay the foundation stone of a new hospital at Aldershot on Tuesday, the 28th inst., at 4.30. Donation purses containing not less than £3 3s. will be presented. The site has been given by Major Newcombe, who initiated the scheme of providing a hospital for the sick poor of Aldershot.

Speaking of street collections for benevolent objects at the last weekly meeting of the London County Council, the *Daily Telegraph* reports that Sir Arthur Arnold said that when the Hospital Saturday Fund started, he suggested the adoption of the system he had seen in Dresden in 1870, when ladies sat in the streets and received alms for the wounded in the Franco-German war. The development of the system would, he thought, prove fatal to the original method. The receipt of alms, which was the modest and appropriate commencement, had now been transformed into continual begging, injurious, he feared, to the young women and girls so engaged, offensive to passengers in the streets, and even at times obstructive to traffic on the footways. As he was responsible for the original suggestion, he took this opportunity of withdrawing his approval from its subsequent and extreme development, and of expressing his conviction that if this perpetual begging, this system of thrusting money-boxes in the faces of passers-by in the streets for all sorts of causes—in every case nominally admirable, but in some cases possibly the cloak of fraud and imposture—was to continue it must lead to prosecution and prevention by the police. A vote of thanks was passed to Sir Arthur for his address, which it was agreed to print in the Council's minutes.

We welcome the co-operation in our crusade against street collections of our able contemporary, the *Daily Mail*, the phenomenal success of which is deservedly due to the enterprise and ability of its conductors. It is matter for congratulation that Mr. W. G. Bunn, the able secretary of the Metropolitan Hospital Saturday Fund, has had the courage to declare, as the result of his experience, that he is personally disposed to acquiesce in the stoppage of street collections altogether unless a short Act of Parliament is passed giving the Home Office or the police power to authorise street collections under certain conditions. Mr. Bunn states that he is hopeful that a considerable proportion of any income that might be sacrificed by the discontinuance of street collections would be made up in another way. There can be no doubt that the amount of public sympathy and the additional hospital support which would accrue to the Metropolitan Hospital Saturday Fund by the abolition of the street collections would ensure a rapid extension of the fund, and that is why we are so anxious for its council to determine to abolish street collections altogether. It is ludicrous to state, as a provincial contemporary maintains, that it is taking a low view of humanity to suggest that any charitable man will withhold an intended benefaction because he is badgered in the streets by the collectors. The propounder of this view can have had no experience of the importunities, obstructive tactics, and persistence of the many street collectors who add to the burden of life in London at the present time.

